# Combined effects of earthworms and *Bacillus* spp. enhance soil ecosystem multifunctionality and reshape microbial communities

**DOI:** 10.3389/fmicb.2026.1799265

**Published:** 2026-04-15

**Authors:** Yuanye Xiao, Menghao Zhang, Hesen Zhong, Cevin Tibihenda, Xinyu Li, My Dung Jusselme, Qi Chao, Runqian Mao, Chi Zhang

**Affiliations:** 1 College of Natural Resources and Environment, South China Agricultural University, Guangzhou, China; 2 Tanzania Agricultural Research Institute, Dodoma, Tanzania; 3 LEESU, Univ Paris Est Creteil, ENPC, Institut Polytechnique de Paris, Creteil, France; 4 Institute of Zoology, Guangdong Academy of Sciences, Guangzhou, China

**Keywords:** earthworm, *Bacillus* spp., tobacco, soil ecosystem multifunctionality, microbial community structure

## Abstract

Long-term physicochemical agricultural management in tobacco systems has impaired the soil ecosystem multifunctionality, disrupted the soil microbial community structure, and affected the long-term health of farmland ecosystems. As key beneficial soil macrofauna and bacteria, earthworms and *Bacillus* spp., respectively, contribute independently to the improvement of soil quality and enhance plant growth. However, their synergistic effects on soil ecosystem multifunctionality and microbial communities remain unclear, particularly in tobacco systems. Therefore, we tested their individual and interactive effects in a tobacco pot experiment with four treatments: control (CK), earthworm inoculation alone (E), *Bacillus* spp. inoculation alone (B), and co-inoculation of earthworms and *Bacillus* spp. (EB). Earthworm inoculation alone significantly increased soil alkaline-hydrolyzable nitrogen and total nitrogen contents, as well as catalase activity, while co-inoculation further enhanced alkaline-hydrolyzable nitrogen, available phosphorus, and catalase activity. Earthworm activity, whether applied individually or in combination with *Bacillus* spp., significantly altered the β-diversity of soil bacterial communities. Both *Bacillus* spp. and earthworm inoculation independently increased the relative abundance of Actinobacteriota and decreased that of *Fusarium*. Moreover, earthworm inoculation alone significantly increased the relative abundance of *Penicillium* and *Mortierella*, whereas co-inoculation significantly increased the relative abundance of Proteobacteria, Bacteroidota, and Verrucomicrobiota. Earthworm inoculation alone and co-inoculation significantly enhanced soil ecosystem multifunctionality. In addition, all biotic treatments (B, E, EB) promoted the complexity of bacterial and fungal co-occurrence networks. Random forest analysis showed that catalase activity and total nitrogen were the key factors affecting soil ecosystem multifunctionality; available phosphorus was the main factor influencing soil bacterial network complexity; and dissolved organic carbon and total nitrogen were the key factors affecting fungal network complexity. In summary, this study demonstrates that earthworms are the core drivers of enhanced soil ecosystem multifunctionality and microbial community structure in tobacco systems, while *Bacillus* spp. exert synergistic effects by strengthening the ecological foundation established by earthworms.

## 1 Introduction

As a globally cultivated and economically important crop, tobacco plays a crucial role in supplying raw materials to various sectors, including pharmaceuticals, chemical industries, and agriculture (Qiu et al., 2023; Han et al., 2025). According to 2024 statistical data, China’s tobacco leaf production reached approximately 2.256 million metric tons, cultivated across a total area of 1.074 million hectares, a level of output that is largely sustained by long-term intensive cultivation (National Bureau of Statistics, 2025). However, such intensification has led to increasingly severe soil degradation, frequent occurrence of soil-borne diseases, and declining ecosystem functions (Pérez-Brandán et al., 2014; Xuan et al., 2024; Wu J. et al., 2025). Previous studies have shown that continuous intensive tobacco cropping reduces soil nitrogen, phosphorus, and potassium contents (Xia et al., 2025), contributes to the incidence of soil-borne diseases caused by pathogens such as *Fusarium* spp. and *Ralstonia solanacearum*, and poses serious threats to tobacco production (Yuliar et al., 2015; Munkvold, 2017). Although physical and chemical measures, including chemical fertilizer application, soil fumigation, and pesticide use (Reichert et al., 2019; Gullino et al., 2022; Zong et al., 2024; Mwalembe et al., 2025), can partially enhance soil fertility and suppress soil-borne diseases, these practices often damage soil ecosystem multifunctionality and microbial diversity (Van Rijssel et al., 2025).

Biological improvement measures have received increasing attention because of their environmental friendliness and high sustainability potential. Such approaches are essential for maintaining soil ecosystem multifunctionality and microbial diversity. Long et al. (2025) reported that stable soil biological communities play a crucial role in sustaining ecosystem processes. Accordingly, the combined application of beneficial soil organisms, such as earthworms and functional microorganisms, may enhance ecosystem functional and biological diversity, thereby promoting soil quality, plant growth, and disease resistance. Earthworms, as keystone species in soil ecosystems, exert positive effects on soil structure, moisture regulation, nutrient cycling, and crop productivity (Decaëns et al., 1999; Blouin et al., 2013; Bertrand et al., 2015). Meanwhile, *Bacillus* spp., as effective biocontrol agents, not only suppress soil-borne diseases but also promote crop growth (Miljaković et al., 2020). Both earthworms and *Bacillus* spp. contribute to the regulation of soil microbial community structure and composition. Earthworm activity creates microbial hotspots that promote carbon, nitrogen, and silicon cycling, enhance microbial growth, metabolism, and enzyme activity (e.g., carboxymethyl cellulase, hemicellulase, and lignin peroxidase); strengthen interactions between Proteobacteria and Actinobacteria, increase microbial network modularity, and stabilize the rhizosphere bacterial community, thereby improving crop growth (Angst et al., 2022; Jiang et al., 2025; Song et al., 2026). In addition, *Bacillus* spp. can modulate soil pH through the secretion of acidic or alkaline metabolites, creating favorable conditions for their own growth and that of coexisting microorganisms, thereby indirectly enhancing microbial activity and diversity (Wang et al., 2025). Although the individual effects of earthworms and *Bacillus* spp. on soil nutrients, microbial characteristics, and crop production have been well demonstrated, their combined effects in agricultural systems remain poorly understood, particularly in tobacco cultivation. Moreover, the key driving factors and mechanisms through which earthworms and *Bacillus* spp. regulate soil ecosystem multifunctionality and microbial community structure remain unclear.

Soil ecosystem multifunctionality and microbial community structure are widely recognized as important indicators of soil health. Soil ecosystem multifunctionality reflects the integrated functioning of nutrient cycling processes and is positively correlated with crop yield (Deng et al., 2024; Shi et al., 2024). Similarly, robust microbial community structure contributes substantially to soil quality. A large-scale study across 27 European countries demonstrated that microbial community structure is a critical determinant of soil health assessment (Romero et al., 2025). Numerous studies have confirmed that soil properties are closely associated with soil ecosystem multifunctionality and microbial community structure. For instance, a 13-year field experiment showed that available phosphorus, dissolved organic carbon, and electrical conductivity are major determinants of soil ecosystem multifunctionality (Wang et al., 2024). Luo et al. (2018) identified soil pH, total nitrogen, and the activities of nitrogen- and phosphorus-cycling enzymes as key positive correlates of soil ecosystem multifunctionality. Soil microbial community succession is mainly influenced by soil carbon and nitrogen contents, with soil dissolved organic carbon playing a particularly important role (Tang et al., 2023). In addition, enzyme activities such as β-glucosidase, N-acetyl-β-D-glucosaminidase, and xylanase, are critical factors affecting microbial community structure and network complexity, and are closely related to soil organic carbon accumulation and microbial functions (Xu et al., 2026). Therefore, overcoming soil-borne diseases, improving crop productivity, maintaining soil ecosystem functions, and promoting sustainable soil development are urgent challenges in tobacco agriculture.

The present study aims to systematically elucidate the effects and mechanisms by which earthworms and *Bacillus* spp. enhance soil ecosystem multifunctionality and improve microbial community structure. To address these objectives, soil samples were collected from farmland in Shaoguan City, China, with more than 10 years of continuous tobacco cultivation. The native earthworm species *Amynthas aspergillum* and *Bacillus* spp. were introduced to address the following scientific questions: (1) Compared with individual applications, does combined application exert greater effects on soil physical-chemical properties and biological characteristics? (2) How do individual and combined applications affect soil ecosystem multifunctionality? (3) How do individual and combined applications influence soil microbial community structure? This study provides a theoretical basis for improving soil health and promoting the sustainable development of ecological tobacco agriculture.

## 2. Materials and methods

### 2.1 Experimental materials

The test soil was collected from farmland in Shaoguan City, Guangdong Province, China (25°05’58″N, 114°16’47″E) with a history of continuous tobacco cultivation for over 10 years. The soil was sampled at 0–20 cm depth, with sand, silt, and clay contents of 37.40, 39.06, and 23.53%, respectively. All samples were air-dried, mixed homogeneously and then sieved through a 2 mm mesh. The basic physicochemical properties of the soil were as follows: pH 7.68 ± 0.06, organic carbon 10.80 ± 0.34 g⋅kg^–1^, total nitrogen 1.07 ± 0.06 g⋅kg^–1^, alkali-hydrolyzable nitrogen 110.83 ± 2.02 mg⋅kg^–1^, available phosphorus 8.65 ± 2.09 mg⋅kg^–1^, and available potassium 212.67 ± 11.93 mg⋅kg^–1^.

The earthworm species used in this study was *Amynthas aspergillum*, purchased from a commercial breeding base located in Qingyuan City, Guangdong Province, China. Following 1 week of stable acclimatization and cultivation in the test soil, healthy individuals with well-developed clitella (A. *aspergillum*, 11.32 ± 1.32 g⋅worm^–1^) were selected.

The microbial agent used in this study was a fully water-soluble compound *Bacillus* spp. formulation, containing *Bacillus subtilis*, *Bacillus licheniformis*, *Bacillus amyloliquefaciens*, and their metabolites (purchased from Weifang Xingyuan Biotechnology Co., Ltd.). To prepare the inoculation, 15 g of the powder was dissolved in 3 L of water, followed by static activation at 25 °C for 2 h. The activated solution was then inoculated into the soil of the corresponding treatment pots via the root irrigation method at a rate of 150 mL⋅worm^–1^.

The tobacco cultivar used in this study was *Nicotiana tabacum* Yunyan 87. Forty-day-old tobacco seedlings (from germination to transplanting stage) were provided by the Crop Research Institute of the Guangdong Academy of Agricultural Sciences, with one seedling planted per pot. The experimental pots were cylindrical with dimensions of 16 × 14 × 18 cm. Small holes were drilled at the bottom of each pot, which was filled with 3 kg of soil, and soil moisture was maintained at 80% of the field water capacity. Both the top and bottom of each pot were sealed with nylon mesh to prevent earthworms from escaping.

### 2.2 Experimental methods

#### 2.2.1 Experimental design

This study was conducted as a pot experiment in a greenhouse at South China Agricultural University from May 11 to July 6, 2025. Four treatments were established: control (CK), earthworm inoculation alone (E), *Bacillus* spp. inoculation alone (B), and co-inoculation of earthworms and *Bacillus* spp. (EB). Each treatment had 5 replicates, totaling 20 pots. At transplanting time, uniform tobacco seedlings were selected and transplanted into pots. Seven days later, approximately 35 g of mature earthworms were acclimatized and then inoculated into the corresponding treatment pots. Simultaneously, 150 mL of *Bacillus* spp. was inoculated into the soil of the corresponding treatment pots using the root drenching method. Watering was performed at appropriate intervals every 3 days. During the experiment, the greenhouse temperature was maintained at approximately 25°C.

#### 2.2.2 Soil analyses

The basic physicochemical properties of the soil were determined according to the methods described by Page (1982). Soil pH was measured using a pH meter (Leici PHS-3C) with a water-to-soil ratio of 2.5:1, and cation exchange capacity was determined using the ammonium acetate method. Soil organic carbon was measured by the potassium dichromate external heating method, while dissolved organic carbon was extracted using the K_2_SO_4_ extraction method. Total nitrogen was determined by the Kjeldahl method and alkali-hydrolyzable nitrogen was measured using the alkali diffusion method. The available phosphorus was extracted with sodium bicarbonate and determined by the molybdenum-antimony colorimetric method, whereas available potassium was extracted with ammonium acetate and measured by flame atomic absorption spectrometry. Soil peroxidase, polyphenol oxidase, β-glucosidase, and acid phosphatase activities were determined using a 96-well microplate fluorometric assay (Luan et al., 2020). Soil urease activity was measured using the sodium phenate colorimetric method (Li et al., 2024), while soil catalase activity was determined by the KMnO_4_ titration method (Cao et al., 2025).

For soil microbial sequencing, 0.5 g of the sample stored at −80°C was used, and genomic DNA was extracted using the TGuide S96 Magnetic Bead Method Genomic DNA Extraction Kit. The concentration of the extracted nucleic acid was detected using a microplate reader, followed by PCR amplification. The integrity of the PCR products was then assessed via electrophoresis on a 1.8% agarose gel. For bacterial 16S rRNA amplification, the V3-V4 region was targeted using universal primers 338F (5’-ACTTACGGGAGGCAGCA-3’) and 806R (5’-GGACTACHVGGGTWTCTAAT-3’). For fungal amplification, the ITS1 region was targeted using universal primers ITS1F (5’-CTTGGTCATTTAGAGGAAGTAA-3’) and ITS2R (5’-GCTGCGTTCTTCATCGATGC-3’). After library preparation, the quality of the libraries was checked using the Qsep-400 method. Qualified libraries were sequenced on the Illumina NovaSeq 6000 platform. Raw data were quality-filtered using Trimmomatic (version 0.33), followed by primer sequence identification and removal using Cutadapt (version 1.9.1). Paired-end reads were then merged, and chimeras were removed using USEARCH (version 10) with UCHIME (version 8.1), resulting in high-quality sequences for downstream analysis. Sequences were clustered into operational taxonomic units (OTUs) at a 97% similarity threshold using USEARCH (version 10.0), with sequences below 0.005% of the total reads filtered out by default.

#### 2.2.3 Plant analyses

Determination of tobacco agronomic traits: Leaf count was determined using the counting method. Plant height, stem diameter, maximum leaf width, and maximum leaf length were measured using a ruler. The above-ground and below-ground biomass were measured using the weighing method.

Tobacco comprehensive growth index (TCGI): Based on the tobacco agronomic trait data (Supplementary Table S1), the comprehensive sub-index SIn for each sample was calculated using the tobacco comprehensive growth potential evaluation method (Zhang et al., 2025). Subsequently, the SIn values were normalized to a range of 0.1–1.0, ultimately yielding the tobacco comprehensive growth index (Y) for each sample. The calculations were performed according to Equations 1, 2.


$$S\,I\,n=W_{1}\times V_{1}+W_{2}\times V_{2}$$ (1)

Y=0.1+(x-b)/(a-b)×0.9 (2)

In the formula: *W*_1_ and *W*_2_ represent the scores of the first and second principal components, respectively, from the principal component analysis for each treatment; *V*_1_ and *V*_2_ denote the cumulative variance contribution rates of the respective principal components; *x* is the original value of each sub-indicator; *a* is the maximum original value among the sub-indicators for all samples; *b* is the minimum original value among the sub-indicators for all samples. The tobacco comprehensive growth index (*Y*) is used to evaluate the overall growth status of tobacco. A higher index value indicates better tobacco growth performance.

#### 2.2.4 Soil ecosystem multifunctionality

The soil ecosystem multifunctionality was calculated using the averaging method (Maestre et al., 2012). Based on soil physicochemical properties and enzyme activities, Z-scores were first computed for each variable. The soil ecosystem multifunctionality was then derived as the average of the Z-scores across all variables. The Z-score was calculated according to Equation 3.


$$Z_{i\,j}=\left(x_{i\,j}-\mu_{j}\right)/\sigma_{j}$$ (3)

In the formula, *Z*_*ij*_ represents the Z-score of the *j*-th soil ecosystem function for sample *i*; *x*_*ij*_ is the actual measured value of the *j*-th soil function for sample *i*; *μ_*j*_* is the mean value of the *j*-th soil ecosystem function across all samples; and *σ_*j*_* is the standard deviation of the *j*-th soil function across all samples.

The soil ecosystem multifunctionality was calculated according to Equation 4.


$$E\,M\,F_{i}=\overset{N}{\underset{j=1}{\sum }}Z_{i\,j}/N$$ (4)

In the formula, *EMF*_*i*_ represents the Soil Ecosystem Multifunctionality for sample *i*, *Z*_*ij*_ denotes the Z-score of the *j*-th soil ecosystem function for sample *i*, and *N* is the number of ecosystem variables included in the functionality assessment.

#### 2.2.5 Complexity of bacterial-fungal networks

Co-occurrence networks were constructed from bacterial and fungal OTUs with a relative abundance greater than 0.1%. Spearman correlation coefficients between pairwise OTUs were calculated using the “Hmisc” package, with *P*-values adjusted by the Benjamini–Hochberg (BH) method. Network modules were identified using the “WGCNA” package. To reduce network complexity, only significant correlations with | r| ≥ 0.7 and *P* < 0.05 were retained for network construction. Visualization and modular analysis of the networks were performed using Gephi (version 0.10.1). Based on the constructed bacterial and fungal co-occurrence networks, key topological parameters (nodes, edges, clustering coefficients, average degree, network density, and average path length) were calculated using the igraph package in R. The standardized network topological parameters were further integrated into a network complexity index by the averaging method (Sun et al., 2025). It is noteworthy that the average path length was calculated as the inverse of the variables before the index was calculated, to align its direction with other complexity parameters.

### 2.3 Statistical analysis

Data were analyzed using one-way analysis of variance (ANOVA) in SPSS 24.0. Duncan’s test was used to analyze the differences between treatments, and the significance level was set at *P* < 0.05. Using the least squares method, polynomial curve fitting was performed on tobacco growth potential and soil ecosystem multifunctionality in Origin 2024. Data analysis was performed using R software (version 4.5.0). The “VennDiagram” package was used to generate Venn diagrams. Principal Coordinates Analysis (PCoA) and Mantel tests were conducted using the “vegan” and “linkET” packages, respectively. Permutational multivariate analysis of variance (PERMANOVA) was applied to assess differences in soil microbial community structure among treatments. Taxonomic composition bar plots and Linear Discriminant Analysis (LDA) were generated using the Microbial Diversity Analysis Platform (Biomarker Technologies). Random Forest (RF) models were built using the “randomForest” package, and the significance of variable importance was assessed with the “rfPermute” package.

## 3 Results

### 3.1 Effects of different treatments on soil physicochemical properties and enzyme activities

**FIGURE 1:**
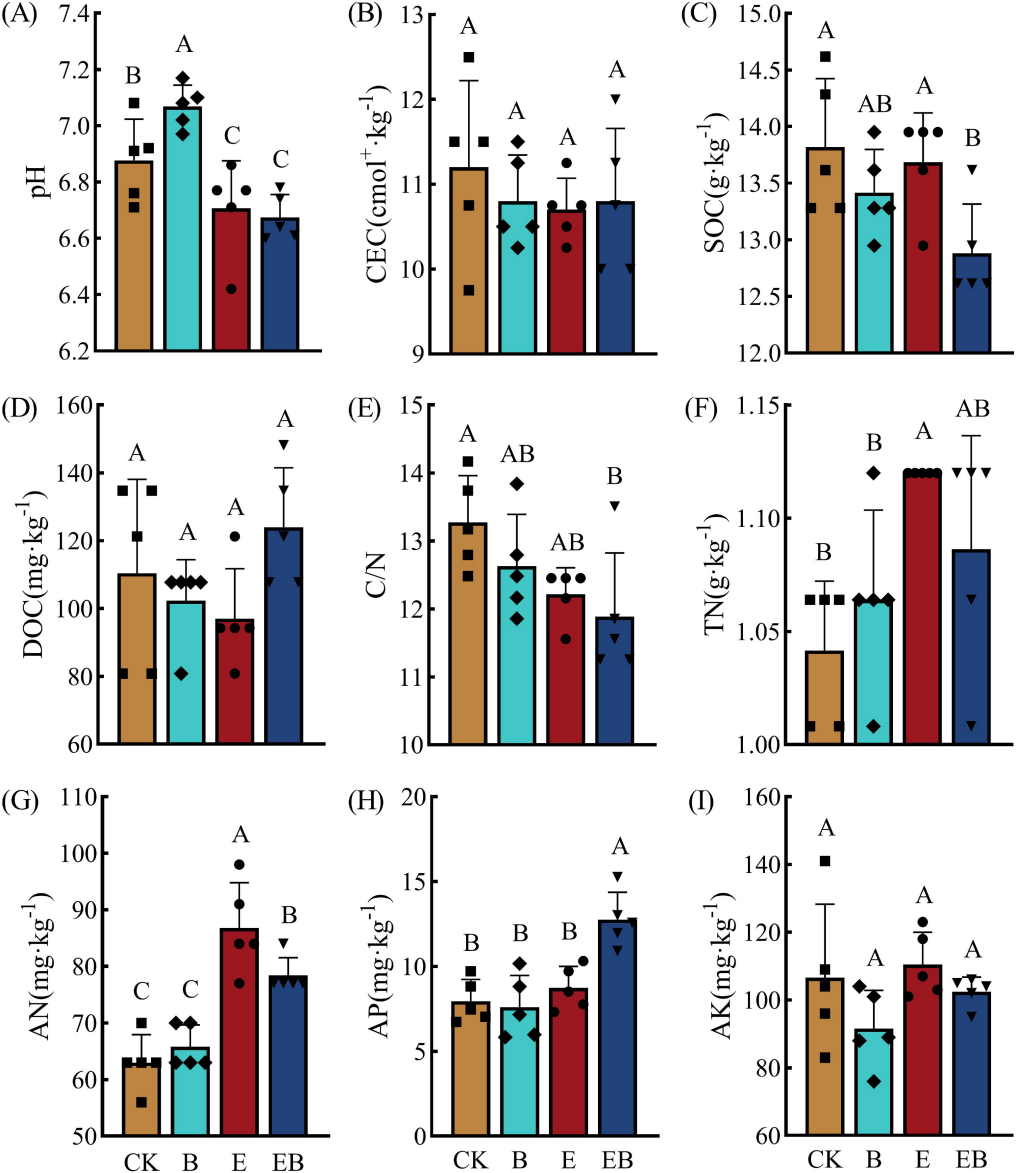
Soil physical and chemical properties under different treatments. Different capital letters indicate significant differences among treatments (*P* < 0.05). **(A)** pH; **(B)** CEC (cation exchange capacity); **(C)** SOC (soil organic carbon); **(D)** DOC (dissolved organic carbon); **(E)** C/N (carbon-to-nitrogen ratio); **(F)** TN (total nitrogen); **(G)** AN (alkali-hydrolyzable nitrogen); **(H)** AP (available phosphorus); **(I)** AK (available potassium).

Compared with CK, treatment B significantly increased soil pH (*P* < 0.05), whereas treatments E and EB significantly decreased soil pH (*P* < 0.05). Treatment E significantly increased AN and TN contents by 37.78 and 7.69%, respectively (*P* < 0.05). Treatment EB significantly reduced SOC content and the C/N ratio, but significantly increased the contents of AN and AP by 24.44 and 60.18%, respectively (*P* < 0.05; Figure 1).

**FIGURE 2:**
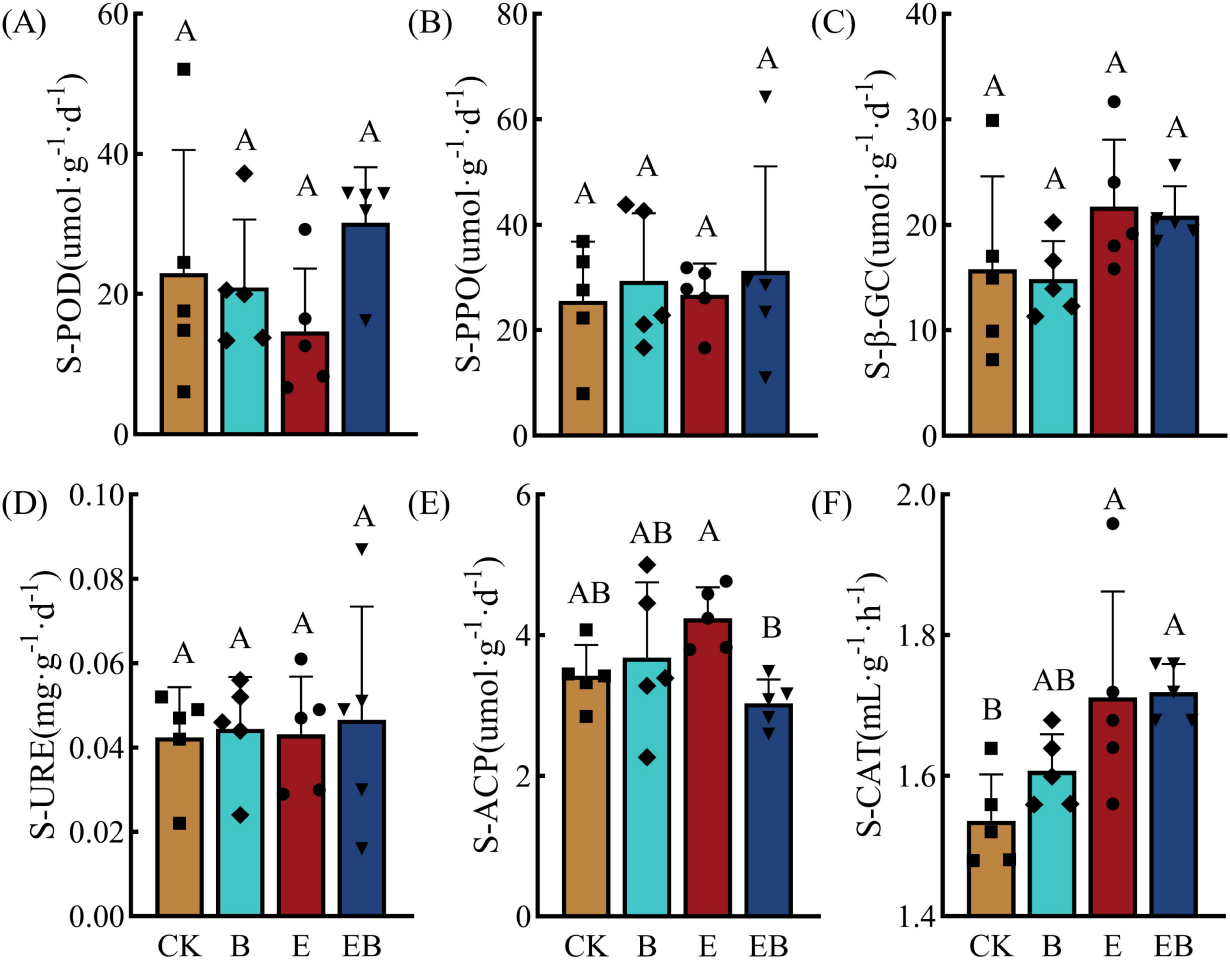
Soil enzyme activity under different treatments. Different capital letters indicate significant differences among treatments (*P* < 0.05). **(A)** S-POD (soil peroxidase); **(B)** S-PPO (soil polyphenol oxidase); **(C)** S-β-GC (soil β-glucosidase); **(D)** S-URE (soil urease); **(E)** S-ACP (soil acid phosphatase); **(F)** S-CAT (soil catalase).

Compared with CK, treatment B showed increasing trends in the activities of S-POD, S-PPO, S-CAT, and S-ACP, although the differences were not statistically significant (*P* > 0.05). Treatment E enhanced the activities of S-PPO, S-CAT, S-β-GC, and S-ACP, with S-ACP activity significantly increasing by 11.04% (*P* < 0.05), whereas the other enzymes did not show significant changes (*P* > 0.05). Treatment EB exhibited an increasing trend in all five soil enzyme activities except S-ACP. In particular, S-CAT activity increased by 11.69% (*P* < 0.05), while changes in the other enzymes were not significant (*P* > 0.05; Figure 2).

### 3.2 Relationship between tobacco comprehensive growth index and soil ecosystem multifunctionality under different treatments

**FIGURE 3:**
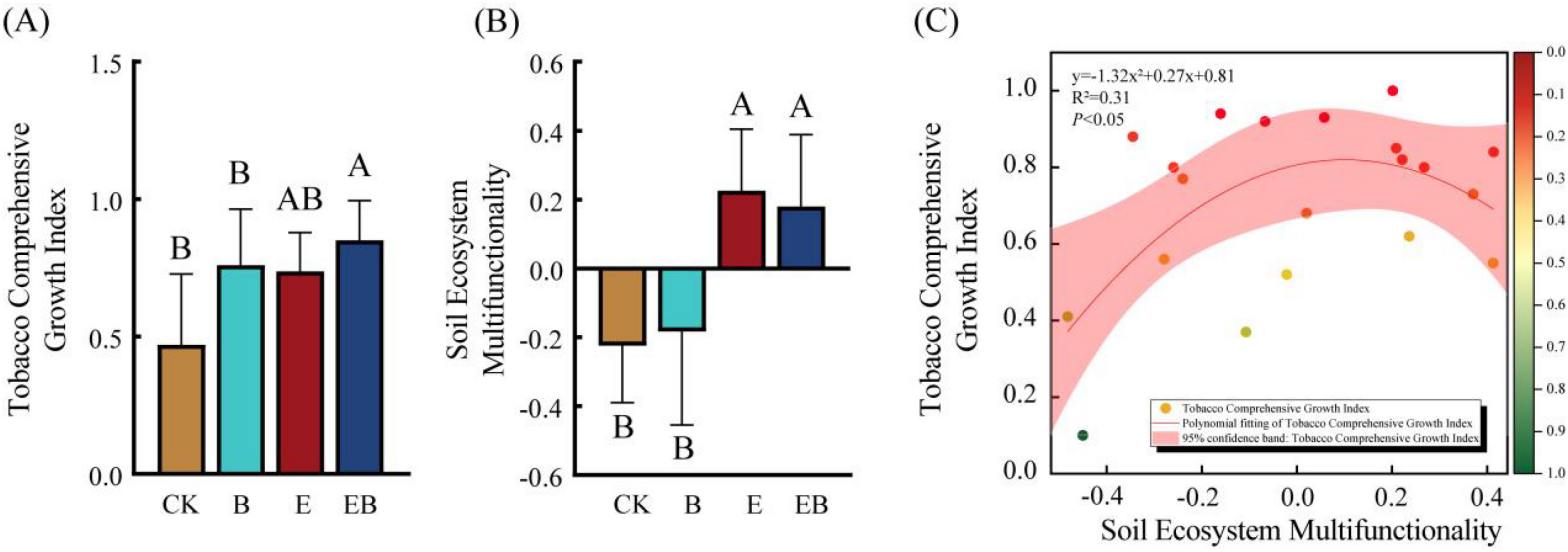
Tobacco comprehensive growth index under different treatments, soil ecosystem functionality, and the relationship between the two Different capital letters indicate significant differences among treatments (*P* < 0.05). **(A)** Tobacco comprehensive growth index. **(B)** Soil ecosystem multifunctionality. **(C)** Polynomial fitting between tobacco comprehensive growth index and soil ecosystem multifunctionality.

Treatments B and EB significantly increased the shoot fresh weight of tobacco (*P* < 0.05). Although treatment E also increased the shoot fresh weight of tobacco, the difference was not statistically significant (*P* > 0.05; Supplementary Table S1). Overall, the addition of earthworms and *Bacillus* spp. improved the TCGI. Compared with CK, treatments B and EB significantly increased TCGI by 61.22 and 80.48%, respectively (*P* < 0.05), whereas treatment E showed an increasing trend without statistical significance (*P* > 0.05; Figure 3A). Treatments E and EB significantly enhanced the EMF (*P* < 0.05), with treatment E showing the highest increase (Figure 3B). The TCGI increased rapidly with the increasing EMF and subsequently tended to stabilize (Figure 3C).

### 3.3 Effects of different treatments on soil microbial communities

#### 3.3.1 Soil microbial community structure

A total of 702 bacterial OTUs and 59 fungal OTUs were shared among the four treatments. For bacteria (Supplementary Figure S1A), all three treatments reduced the number of unique OTUs identified compared with CK. For fungi (Supplementary Figure S1B), treatments B and EB decreased the number of unique soil fungal OTUs, whereas the E treatment increased it, compared with CK.

**FIGURE 4:**
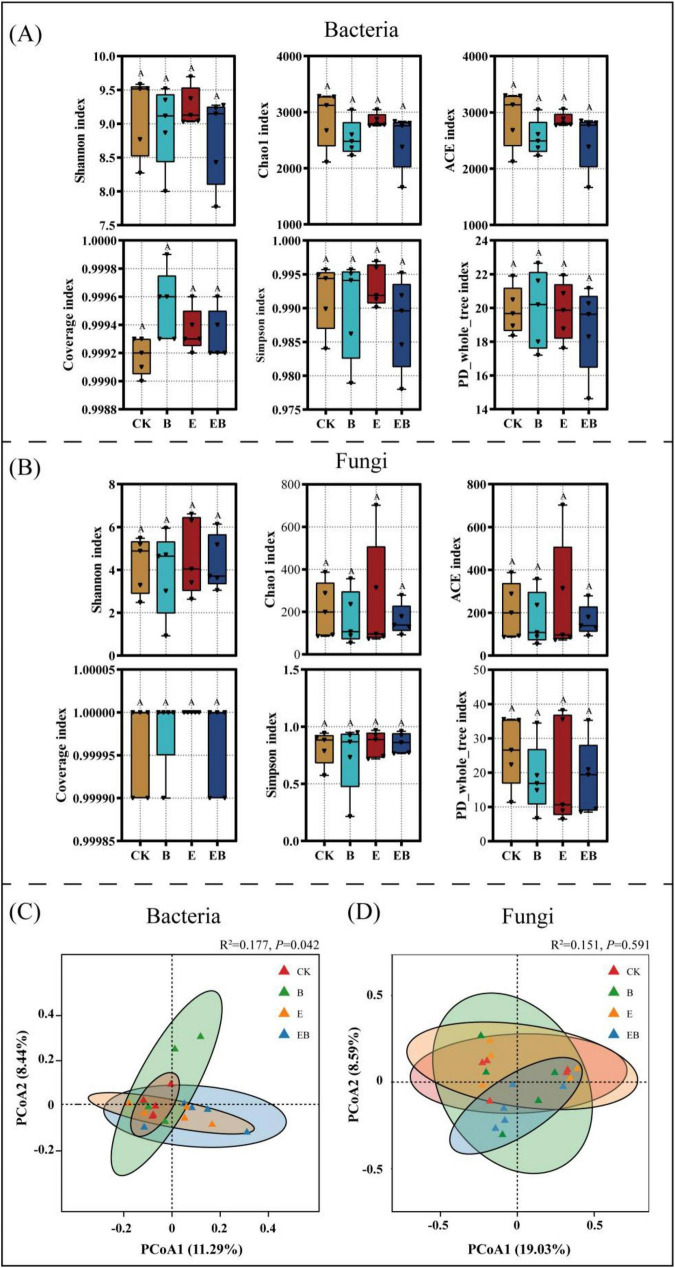
Analysis of soil microbial α-diversity and β-diversity under different treatments. Different capital letters indicate significant differences among treatments (*P* < 0.05). **(A)** Bacterial α-diversity indices. **(B)** Fungal α-diversity indices. **(C)** Bacterial PCoA analysis. **(D)** Fungal PCoA analysis.

The coverage values for bacteria and fungi across all treatments were greater than 0.99, indicating that the sequencing results accurately reflected the true abundance of soil microorganisms. No significant differences in α-diversity of bacteria and fungi were observed among treatments (Figures 4A,B). Principal coordinate analysis (PCoA) based on the Binary-Jaccard distance and Permutational Multivariate Analysis of Variance (PERMANOVA) revealed that treatments significantly affected soil bacterial community composition (*P* < 0.05; Figure 4C); the CK and B treatments formed distinct clusters in space, with clear separation in bacterial community structure, whereas the E and EB treatments formed a single cluster. In contrast, fungal community composition was not significantly affected by treatment (*P* > 0.05; Figure 4D).

#### 3.3.2 Relative abundance of soil microbial communities at the phylum and genus levels

**FIGURE 5:**
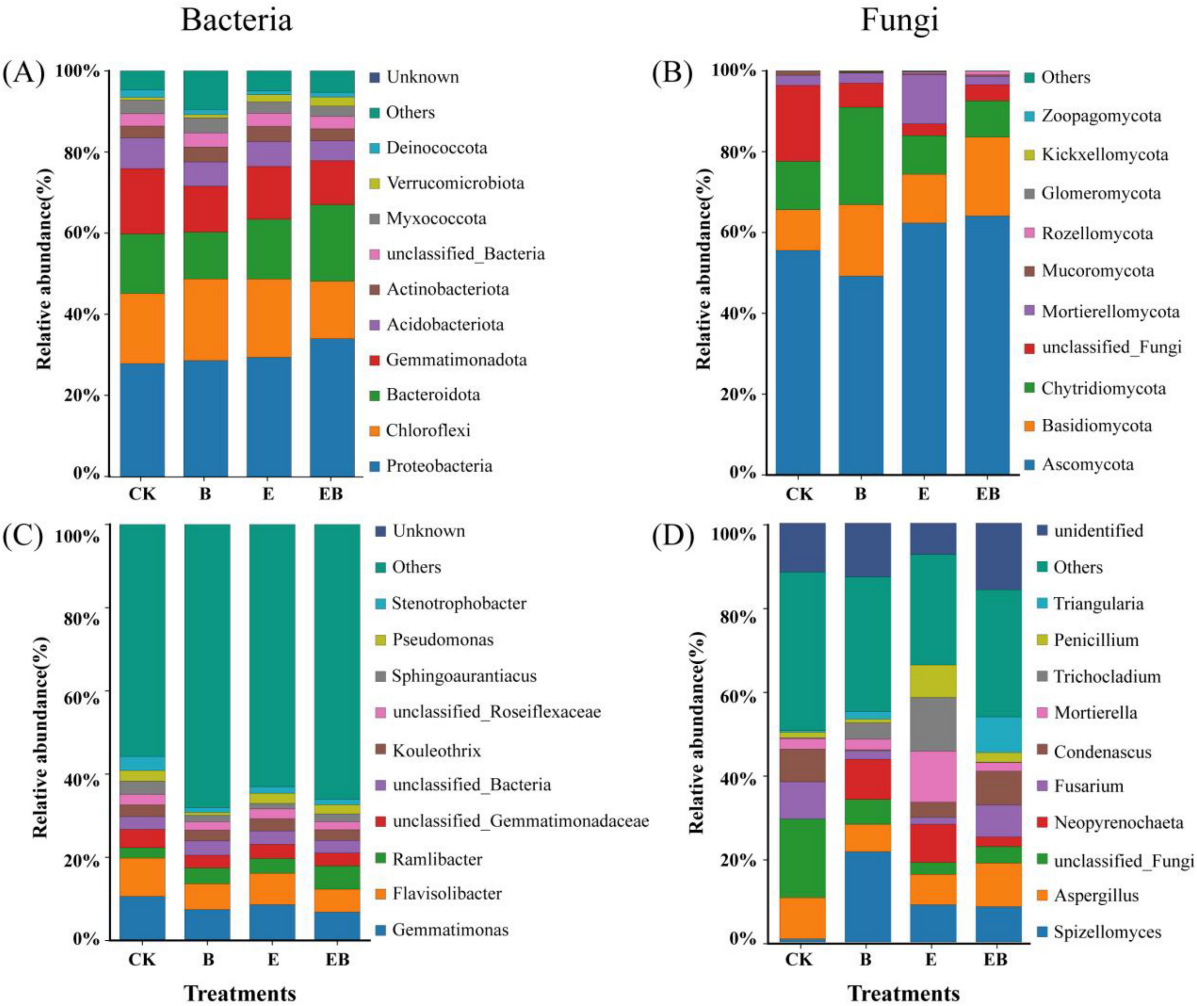
Relative abundance of the top 10 phyla and genera of soil microorganisms under different treatments. **(A,B)** Relative abundance of bacteria and fungi at the phylum level. **(C,D)** Relative abundance of bacteria and fungi at the genus level.

For bacteria (Figures 5A,C), treatment EB increased the relative abundances of Proteobacteria, Bacteroidota, and Verrucomicrobiota, while treatments B and E increased Actinobacteriota. At the genus level, treatments B, E, and EB increased the relative abundance of *Ramlibacter* and decreased those of *Sphingoaurantiacus* and *Stenotrophobacter*.

For fungi (Figures 5B,D), treatments E and EB increased the relative abundances of Ascomycota and Basidiomycota, whereas treatment B increased the relative abundances of Basidiomycota and Chytridiomycota. At the genus level, treatment E increased *Trichocladium*, *Penicillium*, and *Mortierella*. Both treatments B and E reduced the relative abundance of *Fusarium*.

#### 3.3.3 Analysis of differences in soil microbial species abundance

For bacteria (Supplementary Figure S2A), with a Linear Discriminant Analysis (LDA) score exceeding 3.0, treatment EB enriched 18 significantly different bacterial taxa (1 phylum, 1 class, 2 orders, 2 families, 4 genera, and 8 species). In contrast, both treatment E and CK each enriched nine taxa. In particular, treatment B enriched 1 class, 2 families, 3 genera, and 3 species, while CK enriched 1 class, 1 order, 1 family, 1 genus, and 5 species. Treatment E showed the lowest differentiation, with *Phenylobacterium* sp. VITPRS7 being the only significantly enriched species.

For fungi (Supplementary Figure S2B), when the LDA score was greater than 3.0, significantly enriched taxa were detected in all treatments except the control (CK). Treatment EB enriched 6 fungal taxa (1 class, 1 family, 2 genera, 2 species). Treatment B enriched 4 fungal taxa (1 class, 1 family, 2 genera), and treatment E enriched only 2 fungal taxa (2 species).

#### 3.3.4 Co-occurrence network analysis

**FIGURE 6:**
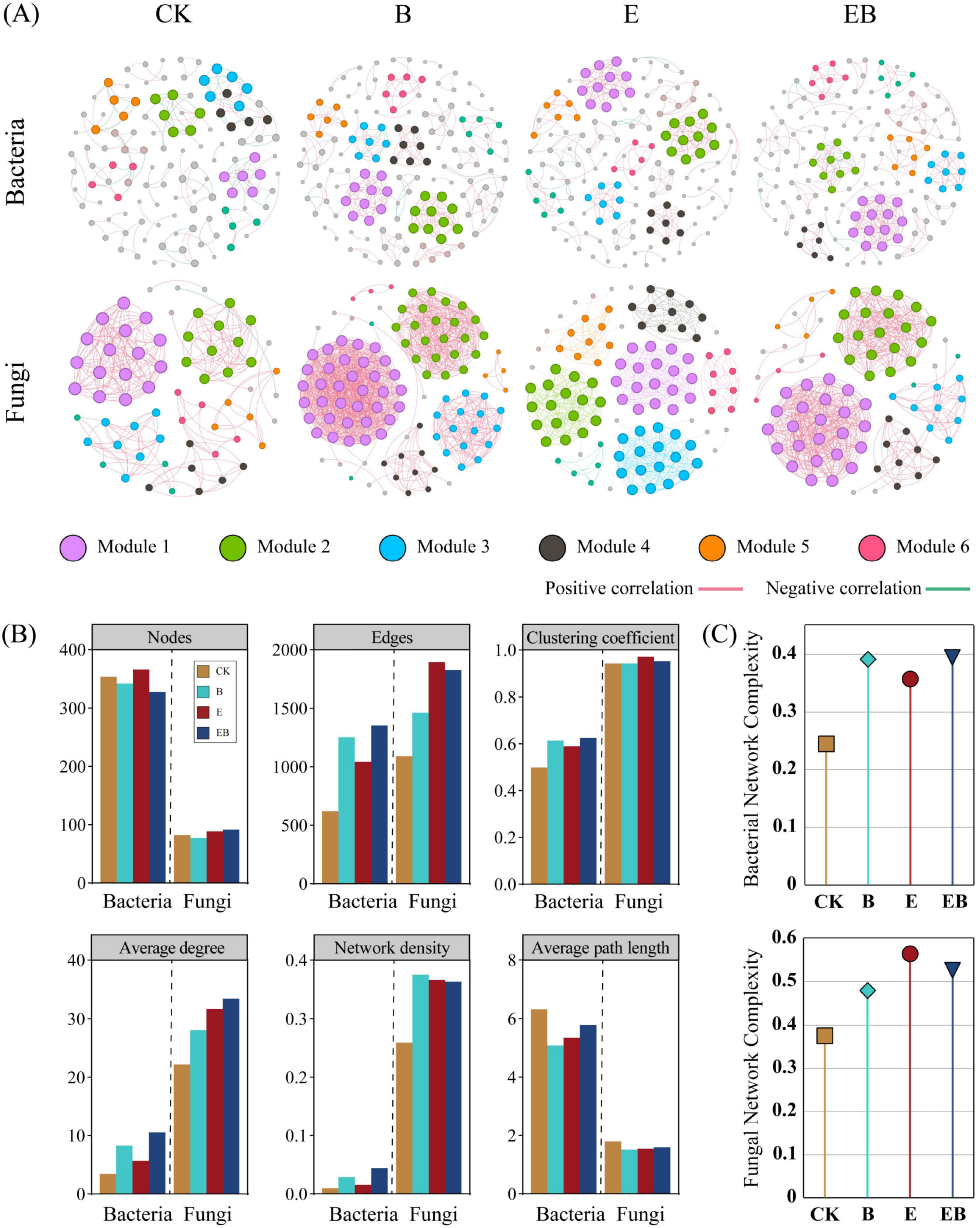
Co-occurrence network analysis of soil bacteria and fungi under different treatments. **(A)** Co-occurrence Networks of Soil Bacteria and Fungi: Nodes in the network represent microbial taxa (OTUs), and the node size is positively correlated with its degree, such that larger nodes indicate taxa with more significant associations with other taxa. Edges represent significant correlations between taxa, with pink indicating positive correlations and green indicating negative correlations. The edge thickness reflects the strength of the correlations. **(B)** Network Topological Parameters of Soil Bacteria and Fungi. **(C)** Complexity of bacterial-fungal networks.

To further elucidate the dynamic changes in soil microbial communities under different treatments, co-occurrence networks were constructed using bacterial and fungal OTUs with a relative abundance greater than 0.1% (Figure 6A). Community complexity and stability were evaluated based on topological parameters. Compared with CK, B, E, and EB all increased the number of edges, clustering coefficients, average degree, and network densities in bacterial and fungal networks, decreased the average path lengths (Figure 6B), and generally increased the complexity of bacterial and fungal community networks (Figure 6C).

### 3.4 Relationship between soil properties and soil microorganisms

**FIGURE 7:**
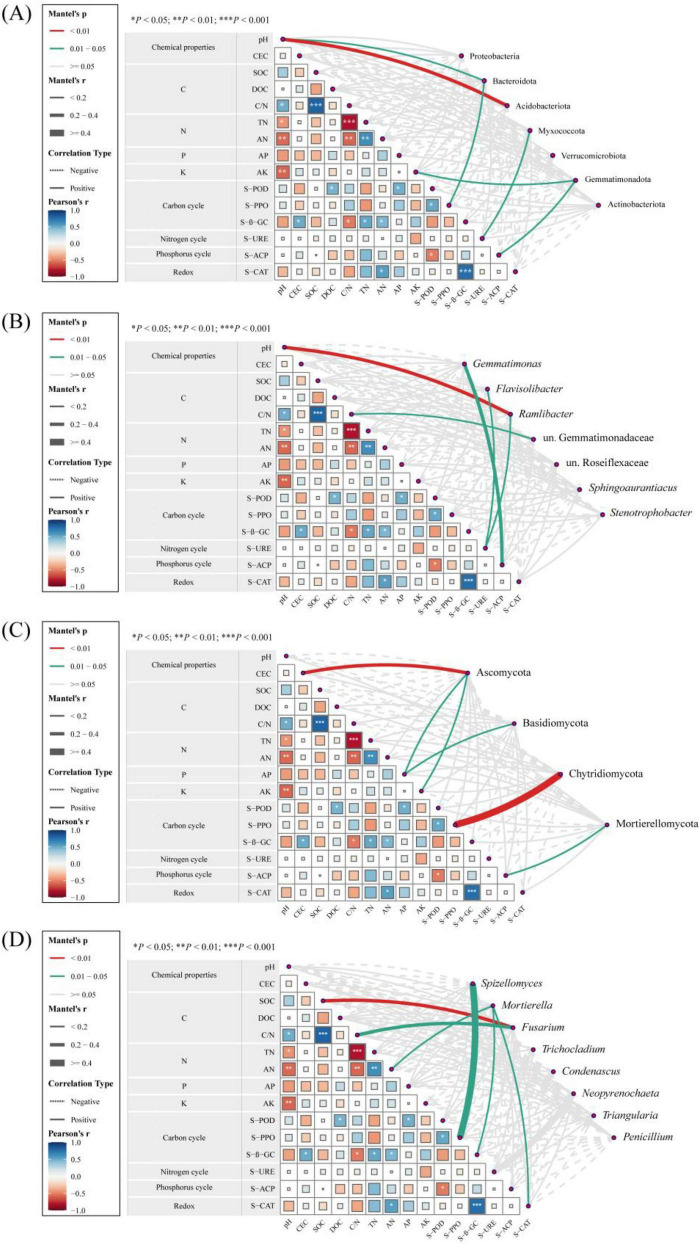
Mantel test analysis of the relationships between soil environmental factors and microorganisms. **(A,B)** Bacterial phyla and bacterial genera. **(C,D)** Fungal phyla and fungal genera. CEC, Cation exchange capacity; SOC, Soil organic carbon; DOC, Dissolved organic carbon; C/N, Carbon- to-nitrogen ratio; TN, Total nitrogen; AN, Alkali-hydrolyzable nitrogen; AP, Available phosphorus; AK, Available potassium; S-POD, Soil peroxidase; S-PPO, Soil polyphenol oxidase; S-β-GC, Soil β-glucosidase; S-URE, Soil urease; S-ACP, Soil acid phosphatase; S-CAT, Soil catalase.

For bacteria (Figures 7A,B), soil pH was significantly positively correlated with Acidobacteriota and *Ramlibacter*. S-ACP was significantly positively correlated with Gemmatimonadota and *Gemmatimonas*. S-URE exhibited significant positive correlations with Myxococcota, *Flavisolibacter*, and *Ramlibacter* (*P* < 0.05).

For fungi (Figures 7C,D), S-PPO showed a significant positive correlation with Chytridiomycota and *Spizellomyces*. CEC, AP, and AK were significantly positively correlated with Ascomycota. AN, S−β−GC, and S-CAT exhibited significant positive correlations with *Mortierella*. SOC and the C/N ratio were significantly positively correlated with *Fusarium* (*P* < 0.05).

### 3.5 Key factors affecting soil ecosystem multifunctionality and microbial community structure

**FIGURE 8:**
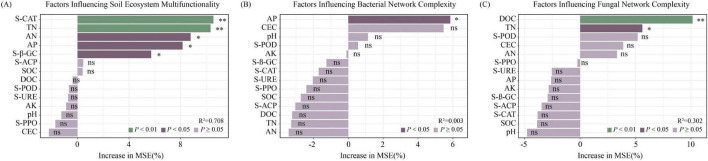
Random forest analysis of the soil ecosystem multifunctionality based on soil physicochemical properties and enzyme activities. **(A)** Variable importance plot of soil ecosystem multifunctionality. **(B)** Variable importance plot of soil bacterial complexity. **(C)** Variable importance plot of soil fungal complexity. CEC, Cation exchange capacity; SOC, Soil organic carbon; DOC, Dissolved organic carbon; TN, Total nitrogen; AN, Alkali-hydrolyzable nitrogen; AP, Available phosphorus; AK, Available potassium; S-POD, Soil peroxidase; S-PPO, Soil polyphenol oxidase; S-β-GC, Soil β-glucosidase; S-URE, Soil urease; S-ACP, Soil acid phosphatase; S-CAT, Soil catalase.

The random forest model indicates that S-CAT, TN, AN, AP, and S-β-GC were identified as key regulators of soil ecosystem multifunctionality, among which S-CAT and TN were the dominant factors affecting EMF (Figure 8A). Regarding soil microbial community, AP was identified as the key factor influencing bacterial communities (Figure 8B); DOC and TN were the key factors shaping fungal communities (Figure 8C).

## 4 Discussion

### 4.1 Individual and combined effects of earthworms and *Bacillus* spp. on soil nutrients and enzyme activities

Both individual and combined inoculation of earthworms and *Bacillus* spp. increased soil nutrient contents through complementary pathways. In this study (Figure 1), inoculation with *Bacillus* spp. (B) significantly increased soil pH, likely due to the secretion of alkaline metabolites (e.g., laurylamine) under acidic conditions, thereby alleviating soil acidification (Cotter and Hill, 2003; Tran et al., 2024). This shift toward neutral pH facilitates the self-regulatory growth of *Bacillus* spp. (Wang et al., 2025). In contrast, earthworm treatments (E and EB) decreased soil pH (Figure 1), which may be attributed to the formation of weak acids in the soil resulting from carbon dioxide and organic acids produced during earthworm metabolism, as well as organic acids secreted by plant roots stimulated by earthworm activities (Wang et al., 2020; Deris and Miroliaei, 2021; Mahohi and Raiesi, 2021). In addition, earthworm inoculation (E) alone significantly increased alkaline-hydrolyzable nitrogen and total nitrogen contents (Figure 1), thereby highlighting the pivotal role of earthworms in regulating nitrogen cycling, which is consistent with the findings of Hao et al. (2024). Meanwhile, the co-inoculation of earthworms and *Bacillus* spp. (EB) not only enhanced soil alkaline-hydrolyzable nitrogen content but also led to a marked increase in available phosphorus content by 60.18% relative to the control (Figure 1), showing strong synergistic effects on phosphorus enrichment. This synergy may result from earthworms fragmenting organic matter and creating favorable microhabitats for microorganisms, thereby facilitating organic nitrogen mineralization (Jiang et al., 2018). *Bacillus* spp., on the other hand, can promote the dissolution and release of insoluble phosphorus by increasing soil phosphatase activity and enriching phosphorus-solubilizing bacteria, thereby increasing plant available phosphorus (Shao et al., 2025).

Both individual and combined treatments also significantly enhanced soil enzyme activities. In fact, soil enzymes are important indicators for evaluating soil quality, are closely associated with organic matter decomposition, nutrient cycling, and microbial activity (Gil-Sotres et al., 2005). As shown in Figure 2, all treatments increased the activities of soil enzymes involved in carbon, nitrogen, and phosphorus cycling to varying degrees. This indicates that a certain level of biological disturbance can effectively activate soil metabolism potential, as confirmed by many previous studies (Tao et al., 2009; Zhang et al., 2016). Catalase (CAT), an important antioxidant enzyme in soil, reflects soil detoxification capacity and stability of soil ecosystems (Elhawat et al., 2025). Earthworm inoculation treatments (E and EB) significantly increased catalase activity (Figure 2), likely through intestinal processing and cast deposition, which alleviate oxidative stress. This is consistent with the results of earthworm cultivation experiments conducted by Li et al. (2022).

### 4.2 Individual and combined effects of earthworms and *Bacillus* spp. on soil ecosystem multifunctionality

In general, the combined inoculation of earthworms and *Bacillus* spp. significantly promoted tobacco growth relative to either inoculant applied alone in our study. *Bacillus* spp. inoculation alone (B) directly enhanced tobacco growth (Figure 3A), consistent with findings of Liang et al. (2025). The growth promotion is mechanistically linked to *Bacillus*-mediated modulation of plant secondary metabolism, particularly the upregulation of flavonoids, which are specialized metabolites in regulating plant growth and development (Pei et al., 2025). Notably, the co-inoculation treatment (EB) produced a significantly greater growth response than individual treatments (E or B) (Figure 3A), indicating strong synergistic effects. This synergy may arise from earthworm-mediated stimulation of root hormone production and soil physical modification through feeding, burrowing, and the release of metabolites, which enhance *Bacillus* spp. colonization, persistence, and functional activity in the rhizosphere (Raja Sekar and Karmegam, 2010).

Earthworm activity also played a central role in improving soil ecosystem multifunctionality (EMF). This ecological index is a comprehensive metric designed to evaluate the overall performance of multiple ecosystem services within a region and provide a holistic understanding of ecosystem dynamics (Zema et al., 2025). In this study, earthworms played a central role in balancing and optimizing EMF (Figure 3B), which is consistent with the findings of many scholars focusing on the ecological functions of earthworms (Liu et al., 2019; Wu D. et al., 2025). A strong quadratic relationship between EMF and TCGI was observed (Figure 3C), indicating that EMF is a predictive indicator for tobacco growth.

Earthworms and *Bacillus* spp. regulate soil ecosystem multifunctionality by modulating key factors, specifically catalase activity and total nitrogen content (Figure 8A). This may be because earthworm activities promote microbial metabolic activity, thereby increasing catalase activity and enhancing the redox capacity and ecological stability of the soil system (Wu et al., 2023). Meanwhile, the synergistic effect of earthworms and *Bacillus* spp. accelerates the turnover of organic matter and the mineralization-immobilization cycle of nitrogen, effectively regulating the size and turnover rate of the total nitrogen pool and providing a dynamic nutrient basis for multiple ecosystem functions (Li Z. et al., 2020). This synergistic regulation mechanism is consistent with the research conclusion of Romero et al. (2025) regarding the enhancement of ecosystem functions by soil biotic interactions.

### 4.3 Individual and combined effects of earthworms and *Bacillus* spp. on soil microbial community structure

The inoculation of earthworms and *Bacillus* spp. induced pronounced restructuring of the bacterial community, characterized by functional shifts rather than taxonomic turnover, alongside selective enrichment of beneficial taxa. Although different treatments did not alter bacterial α diversity (Figure 4B), earthworms, as core drivers, significantly modified bacterial β diversity (Figure 4C) by restructuring the soil microenvironment through burrowing, excretion, and other activities. Consequently, treatments with earthworms (E and EB) formed distinct bacterial communities compared with treatments without earthworms (CK and B) (Vion-Guibert et al., 2024). At the phylum level, both earthworms and *Bacillus* spp., individually enriched Actinobacteriota (Figure 5A), while the combined action enriched beneficial bacteria such as Proteobacteria, Bacteroidetes, and Verrucomicrobota (Figure 5A). The individual actions of earthworms and *Bacillus* spp. primarily enhanced soil organic matter degradation and disease resistance. This is because Actinobacteria can proliferate rapidly by degrading complex, recalcitrant organic matter (Huang et al., 2024). Simultaneously, Actinobacteria produce antibiotics that inhibit other pathogenic bacteria, thereby strengthening soil disease resistance (Shi et al., 2025). The combined action of earthworms and *Bacillus* spp., however, enriches beneficial microbial communities such as the Proteobacteria phylum. These bacteria synergize with *Bacillus* spp. to form metabolic networks characterized by rapid turnover of readily available carbon sources. This process accelerates nutrient cycling rates, like nitrogen and phosphorus, thereby enhancing the soil’s nutrient supply capacity (Shi et al., 2025).

Fungal communities driven by resource inputs and biological interactions exhibit higher functional redundancy and stability, enriching key functional fungi while suppressing the survival of pathogenic fungi. Neither fungal α-diversity nor β-diversity was significantly affected (Figures 4B,D), likely reflecting adaptive regulation within functional groups, thereby maintaining the overall stability of the community structure (Zhong et al., 2025). Inoculation with only earthworms (E) significantly enriched *Penicillium* and *Mortierella*, representing a “golden combination” (Figure 5D). *Penicillium* is a potent decomposer and antibiotic producer (Park et al., 2019; Akaniro et al., 2023); *Mortierella* is an important rhizosphere beneficial fungus (Li F. et al., 2020). The simultaneous enrichment of both highlights the dual advantage of earthworms in promoting organic matter decomposition and enhancing plant health. Additionally, we found that the individual effects of earthworms and *Bacillus* spp. reduced the relative abundance of pathogenic fungi such as *Fusarium* (Figure 5D). This may be because earthworms directly suppress *Fusarium* by recruiting competitive beneficial microbial communities (e.g., the aforementioned *Penicillium* and *Mortierella*), while *Bacillus* spp. itself acts as a biocontrol agent (Tariq et al., 2025), directly exerting an antagonistic effect on soil-borne pathogens.

Both individual and combined inoculations with earthworms and *Bacillus* spp. improved the complexity of microbial community structure (Figure 6C), with available phosphorus, dissolved organic carbon, and total nitrogen identified as the dominant driving factors (Figures 8B,C). Microbial co-occurrence network analysis is an effective approach for examining interspecific interactions among soil microorganisms, evaluating the stability of community structure, and further assessing the functions and health status of soil ecosystems (Berry and Widder, 2014). By promoting phosphorus mineralization, earthworms and *Bacillus* spp. provided key nutrients for phosphorus-limited microbial communities, reduced the direct competition of microorganisms for phosphorus, and instead promoted diverse interactions based on mutual benefit or niche differentiation (Long et al., 2026). Meanwhile, as a high-energy and easily utilizable carbon source, dissolved organic carbon greatly stimulated the metabolic activity and growth of microorganisms, providing an energy basis for establishing more and stronger connections between microorganisms (Hu et al., 2022). The accumulation of total nitrogen pools ensured a continuous nitrogen supply for the synthesis of microbial biomass and metabolic enzymes, thereby stabilizing the long-term structure and functioning of microbial communities (Zhang et al., 2019). Collectively, dissolved organic carbon provided energy, available phosphorus relieved phosphorus limitation, and total nitrogen guaranteed anabolism, and the three together formed a complex network that promoted microbial interactions.

## 5 Conclusion and perspective

This study found that earthworms significantly improve soil fertility, enhance enzyme activities, and reshape soil bacterial community structure. Notably, they enriched beneficial bacterial phyla such as Proteobacteria, Bacteroidota, and Verrucomicrobiota, as well as functional fungal genera including *Penicillium* and *Mortierella*, while suppressing pathogens such as *Fusarium*. In addition, both earthworms and *Bacillus* spp. can promote tobacco growth and improve soil ecosystem multifunctionality. Moreover, their combined application enhances inter-microbial interactions, driving the community structure toward greater complexity and stability. Among these factors, catalase and total nitrogen are the key factors influencing soil ecosystem multifunctionality, available phosphorus is the key factor affecting soil bacterial complexity, and dissolved organic carbon and total nitrogen are the key factors affecting fungal complexity. This study demonstrated that earthworms and *Bacillus* spp. enhance soil ecosystem multifunctionality and improve microbial community structure through pot experiments. However, the field environment is more complex and variable, and the interaction mechanism between the two has yet to be verified for long-term stability. In future studies, long-term, site-specific field experiments are essential to evaluate the ecological adaptability of this model in real farmland environments, thereby providing a practical basis for improving farmland soil health management and sustainable agricultural development.

## Data Availability

The datasets presented in this study can be found in online repositories. The names of the repository/repositories and accession number(s) can be found in the article/Supplementary material.

## References

[B1] Akaniro I. R. Chibuike I. V. Onwujekwe E. C. Gbadamosi F. A. Enyi D. O. Onwe O. N. (2023). *Penicillium* species as chassis for biomanufacturing and environmental sustainability in the modern era: Progress, challenges, and future perspective. *Fungal Biol. Rev.* 46:100326. 10.1016/j.fbr.2023.100326

[B2] Angst G. Frouz J. Van Groenigen J. W. Scheu S. Kögel-Knabner I. Eisenhauer N. (2022). Earthworms as catalysts in the formation and stabilization of soil microbial necromass. *Global Change Biol.* 28 4775–4782. 10.1111/gcb.16208 35543252 PMC9544240

[B3] Berry D. Widder S. (2014). Deciphering microbial interactions and detecting keystone species with co-occurrence networks. *Front. Microbiol.* 5:219. 10.3389/fmicb.2014.00219 24904535 PMC4033041

[B4] Bertrand M. Barot S. Blouin M. Whalen J. De Oliveira T. Roger-Estrade J. (2015). Earthworm services for cropping systems. A review. *Agron. Sustain. Dev.* 35 553–567. 10.1007/s13593-014-0269-7

[B5] Blouin M. Hodson M. E. Delgado E. A. Baker G. Brussaard L. Butt K. R. et al. (2013). A review of earthworm impact on soil function and ecosystem services. *Eur. J. Soil Sci.* 64 161–182. 10.1111/ejss.12025

[B6] Cao X. Zheng H. Wang Y. Geng W. Zi Q. Feng Y. (2025). Enhancing alfalfa yield via micro-nano bubble oxygenation in subsurface drip irrigation: Rhizosphere mechanisms unveiled by structural equation modeling. *Front. Plant Sci.* 16:1702208. 10.3389/fpls.2025.1702208 41341310 PMC12670129

[B7] Cotter P. D. Hill C. (2003). Surviving the acid test: Responses of gram-positive bacteria to low pH. *Microbiol. Mol. Biol. Rev.* 67 429–453. 10.1128/MMBR.67.3.429-453.2003 12966143 PMC193868

[B8] Decaëns T. Rangel A. F. Asakawa N. Thomas R. J. (1999). Carbon and nitrogen dynamics in ageing earthworm casts in grasslands of the eastern plains of Colombia. *Biol. Fertil. Soils* 30 20–28. 10.1007/s003740050582

[B9] Deng H. Ma X. Liu Z. Hu H. Di H. J. Liu Y. et al. (2024). Soil ecosystem multifunctionality is strongly linked with crop yield after four decades chemical fertilization in black soil. *Agric. Ecosyst. Environ.* 368:109007. 10.1016/j.agee.2024.109007

[B10] Deris M. Miroliaei M. R. (2021). Toxic hydrocarbon removal of contaminated soil using eisenia fetida with response surface methodology. *Adv. Environ. Technol.* 7:15. 10.22104/aet.2021.5128.1390

[B11] Elhawat N. Domokos-Szabolcsy É. Veres S. Fári M. G. Alshaal T. (2025). Five-year impacts of biomass crop monoculture on soil enzyme activity, nitrogen pools, and other soil health indicators. *Biomass Bioenergy* 201:107856. 10.1016/j.biombioe.2025.107856

[B12] Gil-Sotres F. Trasar-Cepeda C. Leirós M. C. Seoane S. (2005). Different approaches to evaluating soil quality using biochemical properties. *Soil Biol. Biochem.* 37 877–887. 10.1016/j.soilbio.2004.10.003

[B13] Gullino M. L. Garibaldi A. Gamliel A. Katan J. (2022). Soil disinfestation: From soil treatment to soil and plant health. *Plant Dis.* 106 1541–1554. 10.1094/PDIS-09-21-2023-FE 34978872

[B14] Han S. Yang Y. Xiong S. Zheng H. Yan M. Yang Y. et al. (2025). Plant-microbiome interactions suppress *Fusarium* wilt by enriching beneficial *Aspergillus* in the tobacco rhizosphere. *Environ. Microbiome* 20 159–170. 10.1186/s40793-025-00836-w 41423687 PMC12750933

[B15] Hao R. Wu Y. Di H. Chen Y. Cheng W. Hu R. et al. (2024). Elucidating the role of earthworms on the fate of fertilizer N with synthetic and organic fertilizer application. *Geoderma* 452:117106. 10.1016/j.geoderma.2024.117106

[B16] Hu A. Choi M. Tanentzap A. J. Liu J. Jang K.-S. Lennon J. T. et al. (2022). Ecological networks of dissolved organic matter and microorganisms under global change. *Nat. Commun.* 13:3600. 10.1038/s41467-022-31251-1 35739132 PMC9226077

[B17] Huang Q. Wang B. Shen J. Xu F. Li N. Jia P. et al. (2024). Shifts in C-degradation genes and microbial metabolic activity with vegetation types affected the surface soil organic carbon pool. *Soil Biol. Biochem.* 192:109371. 10.1016/j.soilbio.2024.109371

[B18] Jiang W. Chen R. Song L. Qin L. Xu X. Li X. et al. (2025). From metabolic fingerprints to field solutions: Engineering the Apple rhizosphere microbiome via host-directed *Bacillus* recruitment for sustainable apple replant disease control. *Microbiome* 14:43. 10.1186/s40168-025-02301-9 41437084 PMC12836847

[B19] Jiang Y. Wang J. Muhammad S. Zhou A. Hao R. Wu Y. (2018). How do earthworms affect decomposition of residues with different quality apart from fragmentation and incorporation? *Geoderma* 326 68–75. 10.1016/j.geoderma.2018.04.013

[B20] Li F. Zhang S. Wang Y. Li Y. Li P. Chen L. et al. (2020). Rare fungus, *Mortierella capitata*, promotes crop growth by stimulating primary metabolisms related genes and reshaping rhizosphere bacterial community. *Soil Biol. Biochem.* 151:108017. 10.1016/j.soilbio.2020.108017

[B21] Li J. Xu X. Song L. Na M. Xu S. Zhang J. et al. (2024). Investigating the mechanism of cadmium-tolerant bacterium *Cellulosimicrobium* and ryegrass combined remediation of cadmium-contaminated soil. *Plants* 13:1657. 10.3390/plants13121657 38931089 PMC11207253

[B22] Li W. Zhang P. Qiu H. Van Gestel C. A. M. Peijnenburg W. J. G. M. Cao X. et al. (2022). Commonwealth of soil health: How do earthworms modify the soil microbial responses to CeO_2_ nanoparticles? *Environ. Sci. Technol.* 56 1138–1148. 10.1021/acs.est.1c06592 34964610

[B23] Li Z. Zeng Z. Tian D. Wang J. Wang B. Chen H. Y. H. et al. (2020). Global variations and controlling factors of soil nitrogen turnover rate. *Earth Sci. Rev.* 207:103250. 10.1016/j.earscirev.2020.103250

[B24] Liang Y. Zhang M. Peng B. Zeng X. Li X. Wei J. (2025). Impact of fermented rapeseed cake mixed *Bacillus velezensis* on the bacterial community structure and cultivation of tobacco cultivar K326. *Sci. Rep.* 15:24818. 10.1038/s41598-025-08400-9 40640228 PMC12246417

[B25] Liu T. Chen X. Gong X. Lubbers I. M. Jiang Y. Feng W. et al. (2019). Earthworms coordinate soil biota to improve multiple ecosystem functions. *Curr. Biol.* 29:3420. 10.1016/j.cub.2019.08.045 31587999

[B26] Long X. Li J. Liao X. Wang J. Zhang W. Wang K. et al. (2025). Stable soil biota network enhances soil multifunctionality in agroecosystems. *Global Change Biol.* 31:e70041. 10.1111/gcb.70041 39840664

[B27] Long X. Liao X. Li J. Zhang W. Wang J. Zhang W. et al. (2026). Effects of soil management intensity and soil type on the community assembly and functional potential of soil phosphorus cycling microbes. *Agric. Ecosyst. Environ.* 400:110236. 10.1016/j.agee.2026.110236

[B28] Luan H. Gao W. Huang S. Tang J. Li M. Zhang H. et al. (2020). Substitution of manure for chemical fertilizer affects soil microbial community diversity, structure and function in greenhouse vegetable production systems. *PLoS One* 15:e0214041. 10.1371/journal.pone.0214041 32084129 PMC7034837

[B29] Luo G. Rensing C. Chen H. Liu M. Wang M. Guo S. et al. (2018). Deciphering the associations between soil microbial diversity and ecosystem multifunctionality driven by long-term fertilization management. *Funct. Ecol.* 32 1103–1116. 10.1111/1365-2435.13039

[B30] Maestre F. T. Quero J. L. Gotelli N. J. Escudero A. Ochoa V. Delgado-Baquerizo M. et al. (2012). Plant species richness and ecosystem multifunctionality in global drylands. *Science* 335 214–218. 10.1126/science.1215442 22246775 PMC3558739

[B31] Mahohi A. Raiesi F. (2021). The performance of mycorrhizae, rhizobacteria, and earthworms to improve bermuda grass (*Cynodon dactylon*) growth and Pb uptake in a Pb-contaminated soil. *Environ. Sci. Pollut. Res.* 28 3019–3034. 10.1007/s11356-020-10636-z 32895795

[B32] Miljaković D. Marinković J. Balešević-Tubić S. (2020). The significance of *Bacillus* spp. in disease suppression and growth promotion of field and vegetable crops. *Microorganisms* 8:1037. 10.3390/microorganisms8071037 32668676 PMC7409232

[B33] Munkvold G. P. (2017). “Fusarium species and their associated mycotoxins,” in *Mycotoxigenic Fungi*, eds Moretti A. Susca A. (New York, NY: Springer), 51–106. 10.1007/978-1-4939-6707-0_4 27924531

[B34] Mwalembe D. S. Massawe B. H. J. Nassary E. K. (2025). Pedological characterization of soils under tobacco cultivation: Insights from Sikonge, Uyui and Tabora districts in Tabora Region, Tanzania. *Front. Agron.* 7:1456015. 10.3389/fagro.2025.1456015

[B35] National Bureau of Statistics (2025). *China Statistical Yearbook 2025.* Available online at: https://www.stats.gov.cn/english/ (accessed January 12, 2026).

[B36] Page A. L. (ed.) (1982). *Methods of Soil Analysis: Part 2 Chemical and Microbiological Properties*, 1st Edn. Madison, WI: Wiley. 10.2134/agronmonogr9.2.2ed

[B37] Park M. S. Oh S.-Y. Fong J. J. Houbraken J. Lim Y. W. (2019). The diversity and ecological roles of *Penicillium* in intertidal zones. *Sci. Rep.* 9:13540. 10.1038/s41598-019-49966-5 31537866 PMC6753150

[B38] Pei S. Yue D. Yaoying Y. Zijin H. Zhongmei Z. Xiaoxiang Y. et al. (2025). Elucidating the growth-promoting factors of *Bacillus amyloliquefaciens* on rapeseed through metabolomics. *BMC Plant Biol.* 25:1507. 10.1186/s12870-025-07538-y 41193968 PMC12587698

[B39] Pérez-Brandán C. Huidobro J. Grümberg B. Scandiani M. M. Luque A. G. Meriles J. M. et al. (2014). Soybean fungal soil-borne diseases: A parameter for measuring the effect of agricultural intensification on soil health. *Can. J. Microbiol.* 60 73–84. 10.1139/cjm-2013-0792 24498984

[B40] Qiu R. Li C. Li X. Zhang Y. Liu C. Li C. et al. (2023). First report of *Fusarium sacchari* causing root rot of tobacco (*Nicotiana tabacum* L.) in China. *Crop Prot.* 174:106437. 10.1016/j.cropro.2023.106437

[B41] Raja Sekar K. Karmegam N. (2010). Earthworm casts as an alternate carrier material for biofertilizers: Assessment of endurance and viability of *Azotobacter chroococcum*, *Bacillus megaterium* and *Rhizobium leguminosarum*. *Sci. Hortic.* 124 286–289. 10.1016/j.scienta.2010.01.002

[B42] Reichert J. M. Pellegrini A. Rodrigues M. F. Tiecher T. Dos Santos D. R. (2019). Impact of tobacco management practices on soil, water and nutrients losses in steeplands with shallow soil. *Catena* 183:104215. 10.1016/j.catena.2019.104215

[B43] Romero F. Labouyrie M. Orgiazzi A. Ballabio C. Panagos P. Jones A. et al. (2025). The soil microbiome as an indicator of ecosystem multifunctionality in European soils. *Nat. Commun.* 17 705–733. 10.1038/s41467-025-67353-9 41390845 PMC12820116

[B44] Shao D. He Y. Zhai Y. Yang X. Guo Z. Tan J. et al. (2025). Mechanisms of tomato growth promotion in three soils after applying *Bacillus* combinations. *Soil Tillage Res.* 249:106477. 10.1016/j.still.2025.106477

[B45] Shi G. Li H. Fu Q. Li T. Hou R. Chen Q. et al. (2024). Effects of biochar and compost on the abundant and rare microbial communities assembly and multifunctionality in pesticide-contaminated soil under freeze-thaw cycles. *Environ. Pollut.* 362:125003. 10.1016/j.envpol.2024.125003 39307339

[B46] Shi Y. Li T. Zheng L. Jing X. Hussain H. A. Zhang Q. (2025). Enhancing soil multifunctionality through restoring erosion environment and microbial functions combined with organic manure and straw mulching. *Agric. Ecosyst. Environ.* 383:109515. 10.1016/j.agee.2025.109515

[B47] Song Z. Ling L. Li J. Sun Y. Luo Y. Zhang K. et al. (2026). Earthworms modulate soil microbiota to enhance straw degradation and enrich wheat-available nutrients. *Appl. Soil Ecol.* 217:106626. 10.1016/j.apsoil.2025.106626

[B48] Sun S. Su H. Rao Q. Chen J. Qin Y. Peng Y. et al. (2025). Beyond biodiversity: The more essential role of multi-trophic network complexity for predicting ecosystem multifunctionality under multiple stressors. *Water Res.* 286:124216. 10.1016/j.watres.2025.124216 40683052

[B49] Tang S. Ma Q. Marsden K. A. Chadwick D. R. Luo Y. Kuzyakov Y. et al. (2023). Microbial community succession in soil is mainly driven by carbon and nitrogen contents rather than phosphorus and sulphur contents. *Soil Biol. Biochem.* 180:109019. 10.1016/j.soilbio.2023.109019

[B50] Tao J. Griffiths B. Zhang S. Chen X. Liu M. Hu F. et al. (2009). Effects of earthworms on soil enzyme activity in an organic residue amended rice–wheat rotation agro-ecosystem. *Appl. Soil Ecol.* 42 221–226. 10.1016/j.apsoil.2009.04.003

[B51] Tariq H. Subramanian S. Geitmann A. Smith D. L. (2025). *Bacillus* and *Paenibacillus* as plant growth-promoting bacteria in soybean and cannabis. *Front. Plant Sci.* 16:1529859. 10.3389/fpls.2025.1529859 40525084 PMC12169014

[B52] Tran P. Lander S. M. Prindle A. (2024). Active pH regulation facilitates *Bacillus subtilis* biofilm development in a minimally buffered environment. *mBio* 15:e03387-23. 10.1128/mbio.03387-23 38349175 PMC10936434

[B53] Van Rijssel S. Q. Koorneef G. J. Veen G. F. Pulleman M. M. De Goede R. G. M. Comans R. N. J. et al. (2025). Conventional and organic farms with more intensive management have lower soil functionality. *Science* 388 410–415. 10.1126/science.adr0211 40273235

[B54] Vion-Guibert L. Capowiez Y. Alavoine G. Besaury L. Delfosse O. Hedde M. et al. (2024). The effects of earthworm species on organic matter transformations and soil microbial communities are only partially related to their bioturbation activity. *Soil Biol. Biochem.* 199:109606. 10.1016/j.soilbio.2024.109606

[B55] Wang C. Ma Y. He W. Kuzyakov Y. Bol R. Chen H. et al. (2024). Soil quality and ecosystem multifunctionality after 13-year of organic and nitrogen fertilization. *Sci. Total Environ.* 931:172789. 10.1016/j.scitotenv.2024.172789 38688368

[B56] Wang G. Wang L. Ma F. You Y. Wang Y. Yang D. (2020). Integration of earthworms and arbuscular mycorrhizal fungi into phytoremediation of cadmium-contaminated soil by solanum nigrum L. *J. Hazard. Mater.* 389:121873. 10.1016/j.jhazmat.2019.121873 31862351

[B57] Wang J. Qiu M. Shen Z. Chen L. Ge Y. (2025). Microbial modulation of soil pH: A self-benefiting mechanism exemplified by *Bacillus*. *Soil Biol. Biochem.* 210:109949. 10.1016/j.soilbio.2025.109949

[B58] Wu D. Du E. Eisenhauer N. Mathieu J. Chu C. (2025). Global engineering effects of soil invertebrates on ecosystem functions. *Nature* 640 120–129. 10.1038/s41586-025-08594-y 39939777

[B59] Wu J. Liu Y. Yu H. Fan F. He X. Zhu Y. et al. (2025). An altruistic rhizo-microbiome strategy in crop-rotation systems for sustainable management of soil-borne diseases. *Plant Commun.* 6:101502. 10.1016/j.xplc.2025.101502 40903899 PMC12546766

[B60] Wu P. Xie M. Clough T. J. Yuan D. Wu S. He X. et al. (2023). Biochar-derived persistent free radicals and reactive oxygen species reduce the potential of biochar to mitigate soil N_2_O emissions by inhibiting *nosZ*. *Soil Biol. Biochem.* 178:108970. 10.1016/j.soilbio.2023.108970

[B61] Xia H. Jiang C. Riaz M. Yu F. Dong Q. Yan Y. et al. (2025). Impacts of continuous cropping on soil fertility, microbial communities, and crop growth under different tobacco varieties in a field study. *Environ. Sci. Eur.* 37 5–18. 10.1186/s12302-024-01037-x

[B62] Xu X. Zhang K. Zhu Z. Wei L. Wang S. Zhang W. et al. (2026). Decoding soil health constraints in regional agroecosystems: Machine learning reveals microbial enzymatic thresholds and drivers. *Soil Till. Res.* 258:107000. 10.1016/j.still.2025.107000

[B63] Xuan P. Ma H. Deng X. Li Y. Tian J. Li J. et al. (2024). Microbiome-mediated alleviation of tobacco replant problem via autotoxin degradation after long-term continuous cropping. *iMeta* 3:e189. 10.1002/imt2.189 38882490 PMC11170962

[B64] Yuliar, Nion Y. A. Toyota K. (2015). Recent trends in control methods for bacterial wilt diseases caused by *Ralstonia solanacearum*. *Microbes Environ.* 30 1–11. 10.1264/jsme2.ME14144 25762345 PMC4356456

[B65] Zema D. A. Carmona-Yáñez M. D. Plaza-Alvarez P. A. Lucas-Borja M. E. (2025). A new index to estimate ecosystem multifunctionality: Theoretical approach and an application to a burned forest of Central Eastern Spain. *J. Environ. Manage.* 373:123655. 10.1016/j.jenvman.2024.123655 39672050

[B66] Zhang C. Mora P. Dai J. Chen X. Giusti-Miller S. Ruiz-Camacho N. et al. (2016). Earthworm and organic amendment effects on microbial activities and metal availability in a contaminated soil from China. *Appl. Soil Ecol.* 104 54–66. 10.1016/j.apsoil.2016.03.006

[B67] Zhang L.-Y. Mai J. Li B.-L. Fei L.-N. Lin J.-C. Lu W.-P. et al. (2025). Comprehensive evaluation and mechanisms of tobacco quality improvement via microbial fermentation. *Ind. Crops Prod.* 236:121931. 10.1016/j.indcrop.2025.121931

[B68] Zhang S. Zheng Q. Noll L. Hu Y. Wanek W. (2019). Environmental effects on soil microbial nitrogen use efficiency are controlled by allocation of organic nitrogen to microbial growth and regulate gross N mineralization. *Soil Biol. Biochem.* 135 304–315. 10.1016/j.soilbio.2019.05.019 31579295 PMC6774787

[B69] Zhong Y. Zhang B. Zhu Y. Shangguan Z. Li T. Deng L. et al. (2025). Shifts in the fungal community promote soil carbon accumulation in microaggregates during long-term secondary succession. *Funct. Ecol.* 39 2029–2043. 10.1111/1365-2435.70076

[B70] Zong D. Zhou Y. Zhou J. Zhao Y. Hu X. Wang T. (2024). Soil microbial community composition by crop type under rotation diversification. *BMC Microbiol.* 24:435–447. 10.1186/s12866-024-03580-2 39462325 PMC11520043

