# Supplementary material for: Combined effects of earthworms and Bacillus spp. enhance soil ecosystem multifunctionality and reshape microbial communities
Source: Front Microbiol. 2026 Apr 15;17:1799265. doi: 10.3389/fmicb.2026.1799265 (PMC13125008; doi:10.3389/fmicb.2026.1799265)
Supplement: Supplementary file 1 [file Data_Sheet_1.pdf]

Supplementary Material

Table list:

Table S1. Tobacco agronomic traits under different treatments

Data are presented as mean ± standard deviation. Different capital letters indicate significant differences among treatments (*P* < 0.05).

Table S1. Tobacco agronomic traits under different treatments

Treatments	Plant Height (cm)	Leaf Number	Maximum Leaf Width (cm)	Maximum Leaf Length (cm)	Shoot Fresh Weight (g)	Root Fresh Weight (g)
CK	55.04±6.17A	10.80±1.30A	14.12±1.63AB	31.88±3.43AB	64.78±10.02B	3.70±0.91A
B	54.20±3.42A	11.40±0.55A	16.04±2.01A	33.32±1.91A	78.20±9.03A	4.64±1.18A
E	60.70±1.57A	11.60±1.34A	13.68±0.72B	29.58±0.93B	73.82±5.98AB	4.24±1.65A
EB	57.20±5.81A	12.40±0.55A	14.16±1.05AB	31.94±1.18AB	81.08±4.47A	4.36±0.53A

Data are presented as mean ± standard deviation. Different capital letters indicate significant differences among treatments ($P < 0.05$).

Figure captions

Figure S1. Venn diagrams of (A) soil bacteria and (B) soil fungi based on OTU classification

Figure S2. LEfSe analysis of (A) soil bacterial and (B) soil fungal communities under different treatments

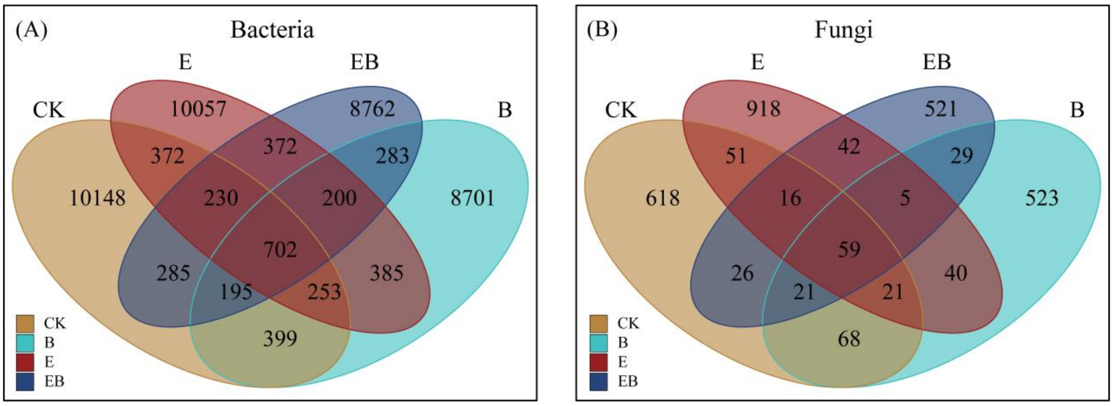

Figure S1. Venn diagrams of (A) soil bacteria and (B) soil fungi based on OTU classification

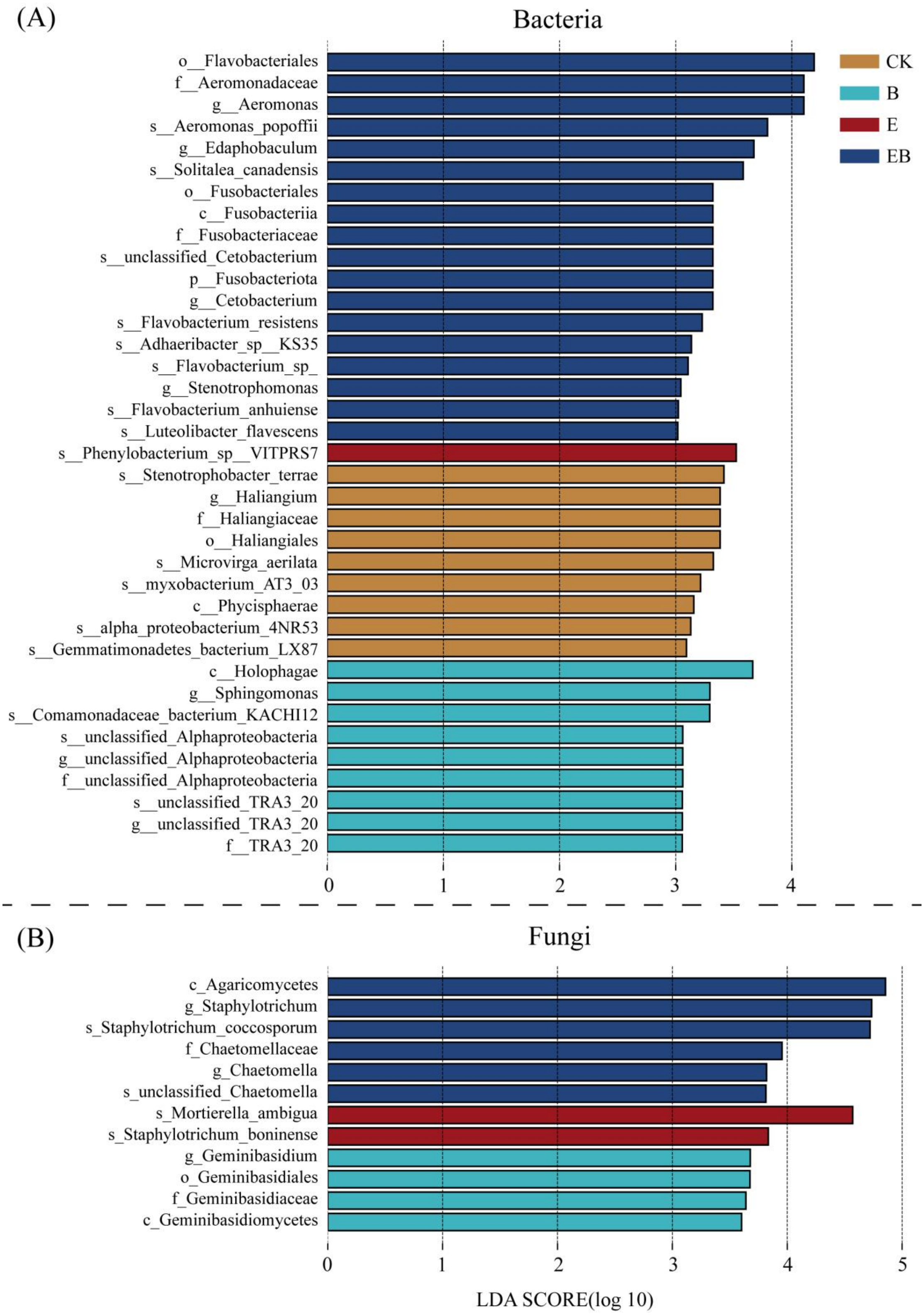

Figure S2. LEfSe analysis of (A) soil bacterial and (B) soil fungal communities under different treatments
